# Secondary fracture prevention in Spanish primary care: results of the PREFRAOS Study

**DOI:** 10.1007/s11657-024-01394-3

**Published:** 2024-05-09

**Authors:** Daniel Martínez-Laguna, Cristina Carbonell Abella, José-Carlos Bastida, Milagros González, Rafael M. Micó-Pérez, Francisco Vargas, Eva Díaz Torres, Laura Canals, on behalf of the PREFRAOS Group

**Affiliations:** 1 Health Center Sant Martí de Provençals, C/ Fluvià 211, Barcelona, Spain; 2 GREMPAL Research Group, IDIAP Jordi Gol, Barcelona, Spain; 3 Health Center Vía Roma, Barcelona, Spain; 4 Health Center of Marín, Pontevedra, Spain; 5 Health Center of Montesa, Madrid, Spain; 6 Health Center of Fontanars Dels Alforins, EAP Ontinyent, Valencia, Spain; 7 Health Center Dr. Guigou, Santa Cruz de Tenerife, Spain; 8 Medical Department, Amgen, Spain; 9 Medical Department, Amgen, Switzerland

**Keywords:** Clinical management, Fragility fracture, Osteoporosis, Primary care, Secondary fracture prevention

## Abstract

**Summary:**

This study demonstrated a large treatment gap in elderly subjects experiencing fragility fracture in Spanish primary care, a low treatment persistence among subjects who do receive treatment, and more than one-quarter having no follow-up visits post-fracture. These data highlight the need to improve secondary fracture prevention in primary care.

**Purpose:**

To describe osteoporosis (OP) treatment patterns and follow-up in subjects with fragility fracture seen in Spanish primary care (PC).

**Methods:**

This observational, retrospective chart review included subjects aged ≥ 70 years listed in the centers' records (November 2018 to March 2020), with ≥ 1 fragility fracture and prior consultation for any reason; subjects who had participated in another study were excluded. Outcomes included OP treatments and follow-up visits post-fragility fracture.

**Results:**

Of 665 subjects included, most (87%) were women; overall mean (SD) age, 82 years. Fewer than two thirds (61%) had received any prior OP treatment (women, 65%; men, 38%); of these, 38% had received > 1 treatment (women, 25%; men, 13%). Among treated subjects, the most frequent first-line treatments were alendronate (43%) and RANKL inhibitor denosumab (22%), with a higher discontinuation rate and shorter treatment duration observed for alendronate (discontinuation, 42% vs 16%; median treatment duration, 2.5 vs 2.1 years). Over one-quarter (26%) of subjects had no follow-up visits post-fragility fracture, with this gap higher in women than men (35% versus 25%). The most common schedule of follow-up visits was yearly (43% of subjects with a fragility fracture), followed by half-yearly (17%) and biennial (10%), with a similar trend in men and women. Most OP treatments were prescribed by PC physicians, other than teriparatide and zoledronate.

**Conclusions:**

Across Spanish PC, we observed a large gap in the treatment and follow-up of elderly subjects experiencing a fragility fracture. Our data highlights the urgent need to improve secondary fracture prevention in PC.

**Supplementary Information:**

The online version contains supplementary material available at 10.1007/s11657-024-01394-3.

## Introduction

Fragility fractures, defined as low energy trauma resulting from a fall from standing height or less, are a sign of underlying osteoporosis (OP), and increase the risk of further fractures [1, 2]. The risk of fragility fractures increases with age and, due to the fall in estrogen levels during the menopause which causes rapid acceleration in bone loss, is higher for women than men [3–5]. Other risk factors include a family history of OP, certain medications (including glucocorticoids), excessive alcohol intake, smoking, low body mass index, calcium and vitamin D deficiency, and sedentarism [6].

In Europe, fragility fractures are the fourth leading cause of chronic disease morbidity after ischemic heart disease, dementia and lung cancer [7], with an estimated prevalence of 6.8% and 22.5% in men and women aged ≥ 50 years, respectively [8]. We previously reported comparable prevalence from the PREFRAOS study of Spanish Primary Care (PC); men, 8.0%; women, 24.1%) [9]. The International Osteoporosis Foundation (IOF) estimate the annual incidence of fragility fractures in the largest five countries of the European Union plus Sweden (EU6) will increase by 23% between 2017 and 2030, from 2.7 million to 3.3 million [8]. Moreover, the total costs associated with fragility fractures in the European Union were estimated to be € 29.6 billion in 2010 and are expected to increase by approximately 54% in 2030 [7]. Similarly, based on existing disease management patterns across 8 European countries, Yeh and colleagues estimated annual fracture rates and associated costs in women aged ≥ 70 years will increase by 44% between 2020 and 2040 (from 1.2 million fractures costing € 12.8 billion to 2.8 million fractures costing € 18.4 billion) [10].

Despite these costs, and guidelines recommending OP treatment in subjects who have sustained a fragility fracture [11–13], OP treatment rates are low and many studies report a treatment gap in subjects at high fracture risk. For example, the IOF reported the treatment gap in 2017 to be 73% in women and 63% in men, with most (women, 89%; men 97%) treatment naïve at the time of their first fracture[8]. Similarly, a cross-sectional study of women aged ≥ 70 years seen in PC across 8 European countries reported an overall treatment gap of 75%, and a higher gap among women without an OP diagnosis (94%) versus those with an OP diagnosis (31%) [14]. Phase A of our study of Spanish PC data previously reported a treatment gap of 39% in men and women aged ≥ 70 years experiencing a fragility fracture [9].

Data reporting secondary fracture prevention by PC physicians is sparse. Our retrospective observational study aimed to estimate the prevalence of fragility fractures in Spanish PC and describe the characteristics, risk factors, co-morbidities, and OP diagnosis and treatment rates in subjects with a fragility fracture. With data regarding the prevalence of fragility fractures and OP diagnosis and treatment rates already published [9]; we describe OP treatment patterns and follow-up in subjects with at least a fragility fracture seen in Spanish PC.

## Methods

### Study design

The observational, retrospective single-country PREvalence of FRAgility fractures and OSteoporosis treatment (PREFRAOS) study was conducted in 30 Spanish PC centers across 14 Spanish Regions. The study design and methodology have been published previously [9].

In brief, the study comprised two phases: Phase A included subjects aged ≥ 70 years listed in the center’s medical records between November 2018 and March 2020; Phase B included subjects from Phase A with at least a recorded fragility fracture and prior consultation at the center for any reason. Fragility fractures were identified using International Classification of Diseases (ICD) codes (ICD-9 and ICD-10) and/or open fields, according to the characteristics of each center’s database. Study outcomes were collected retrospectively from subjects’ medical records. A full list of study outcomes is given in Supplementary Methods Section. Results from Phase A and some results from Phase B were published previously [9]. Herein, we describe OP treatment patterns and follow-up in subjects with a fragility fracture included in Phase B.

This study was conducted according to the standards of the Declaration of Helsinki principles, its later amendments and with Good Clinical Practice guidelines. All subjects provided written or oral informed consent before enrolment.

### Statistical analysis

All analyses were performed using SAS version 9.4 (SAS Institute, Cary, NC, USA). For categorical variables, the frequency and percentage with 95% confidence interval (CI) are presented. For continuous variables, summary statistics included the number of subjects, mean, median, standard deviation (SD) or standard error (SE), 25th percentile (Q1), 75th percentile (Q3), minimum, and maximum.

## Results

### Demographic and clinical characteristics

As reported previously, 665 subjects were included in Phase B with a mean (SD) age of 82 (6.6) years, and most subjects were women and had experienced only one fragility fracture (87% and 72%, respectively) [9]. Mean (SD) age at the time of first fragility fracture was 76 (8.6) years, with the first fracture reported to be related to a fall in over three quarters (78%) of the population. Mean (SD) time from first to second fragility fracture was numerically shorter in men than women (3.4 [4.9] versus 5.3 [4.7] years] (Table 1).

**Table 1:** Characteristics of subjects experiencing a fragility fracture – data from Spanish Primary Care

	Overall (N = 665)	Women (N = 567)	Men (N = 98)
Age at first fragility fracture (years), mean (SD)	76.1 (8.6)	75.7 (8.3)	78.3 (10.0)
Time from first fragility fracture to IC (years), mean (SD)	5.8 (5.4)	6.0 (5.2)	4.8 (6.1)
Time from first to second fragility fracture (years), mean (SD)	5.1 (4.8)	5.3 (4.7)	3.4 (4.9)
First fragility fracture related to a fall (yes), n (%)	517 (77.7)	443 (78.1)	74 (75.5)

*IC* informed consent, *FF* fragility fracture, *SD* standard deviation

### Osteoporosis treatments

**Fig. 1:**
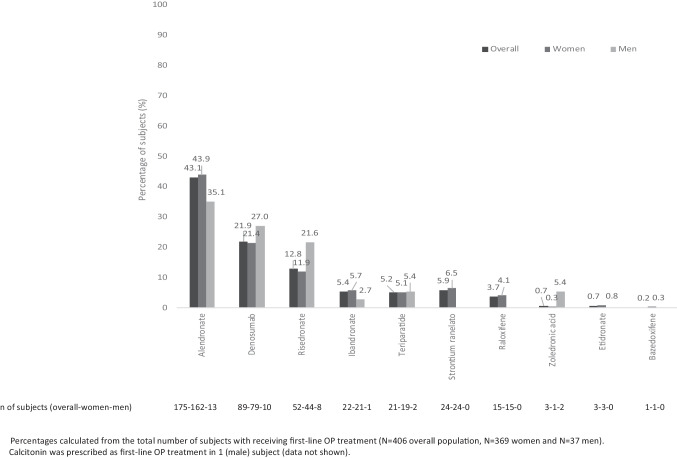
First-line OP treatments in subjects experiencing a fragility fracture—data from Spanish Primary Care

Overall, 61.1% (406/665) of subjects with a fragility fracture had received prior OP treatment and three-quarters (501/665) had received calcium or vitamin D supplements. Treatment rates were higher among women than men (any prior OP treatment, 369/567 [65.1%] vs 37/98 [37.8%]; calcium or vitamin D, 440/567 [77.6%] vs 61/98 [62.2%]). Among subjects who had received prior OP treatment, 37.9% (154/406) had received more than one therapy, with women more likely than men to have received multiple therapies (141/567 [24.9%] vs 13/98 [13.3%]). The most common first-line OP treatments were alendronate (175/406, 43.1%) followed by RANKL inhibitor denosumab (89/406, 21.9%)] (Fig. 1).

At study inclusion, fewer than half (287/665 [43.2%)] of subjects with a fragility fracture were on active OP treatment, with treatment rates higher in women than men (45.7% vs 28.6%) [9] and the most common treatments being denosumab and alendronate (149/287 [51.9%] and 97/287 [33.8%], respectively). Denosumab use was numerically higher in women than in men ([138/259 [53.3%] vs 11/28 [39.3%]) (Table 2).

**Table 2:** Summary of subjects on active OP treatment at the time of study enrolment – data from Spanish Primary Care

	Overall	Women	Men
Subjects not receiving treatment, n (%)^a^	378 (56.8)	308 (54.3)	70 (71.4)
Subjects receiving treatment, n (%)^a^	287 (43.2)	259 (45.7)	28 (28.6)
Denosumab^b^	149 (51.9)	138 (53.3)	11 (39.3)
Alendronate^b^	97 (33.8)	86 (33.2)	11 (39.3)
Alendronate + denosumab^b^	1 (0.3)	1 (0.4)	0 (0.0)
Risedronate^b^	15 (5.2)	12 (4.6)	3 (10.7)
Ibandronate^b^	12 (4.2)	12 (4.6)	0 (0.0)
Teriparatide^b^	9 (3.1)	7 (2.7)	2 (7.1)
Zoledronic acid^b^	3 (1.0)	2 (0.8)	1 (3.6)
Bazedoxifene^b^	1 (0.3)	1 (0.4)	0 (0.0)
Subjects with OP diagnosis receiving treatment, n (%)^c^	347 (79.4)	319 (80.8)	28 (66.7)
Subjects without OP diagnosis receiving treatment, n (%)^d^	59 (25.9)	50 (29.1)	9 (16.1)

^a^Percentages calculated from the total number of subjects (N = 665 overall, N = 567 women and N = 98 men); ^b^Percentages calculated from the number of subjects who received treatment (N = 287 overall, N = 259 women and N = 28 men); ^c^Percentages calculated from the number of subjects with OP diagnosis (N = 437 overall, N = 395 women and N = 42 men) and ^d^Percentages calculated from the number of subjects without OP diagnosis (N = 228 overall, N = 172 women and N = 56 men)

OP treatment rates by OP diagnosis are displayed in Table 2. Compared with subjects without a diagnosis, OP treatment rates (at any time) were higher in subjects with an OP diagnosis (79.4% vs 25.9%). The same trend was observed in women and in men: 80.8% of women and 66.7% of men with an OP diagnosis had received an OP treatment compared with 29.1% of women and 16.1% of men without an OP diagnosis. A higher OP treatment rate was reported in women (with and without OP diagnosis) compared to men.

As reported previously, almost half (298/661 [45.1%]) of all OP treatments prescribed were discontinued, most commonly at the decision of the treating specialist (95/298, 31.9%) or the subject (62/298, 20.8%), or due to tolerability issues (57/298, 19.1%) [9]. A total of 42.3% (96/227) of alendronate prescriptions were discontinued, with a numerically higher discontinuation rate in women than men (94/212 [44.3%] vs. 2/15 [13.3%]). Alendronate was most commonly discontinued at the decision of the treating specialist or the subject (23/96 [24.0%] and 21/96 [21.9%], respectively), or due to tolerability issues (21/96 [21.9%]). Only 15.5% (27/174) of denosumab prescriptions were discontinued, with a numerically lower discontinuation rate in women than men (23/159 [14.5%] vs 4/15 [26.7%]); the most common reason for discontinuation was the subject’s decision (17/27 [63.0%]). Reasons for prescription discontinuations are shown in Table SI1.

Median (Q1, Q3) treatment duration was 2.5 (0.6, 5.3) years for alendronate (higher in women vs men: 2.5 [0.6, 5.6] vs 1.0 [0.4, 4.1]) and 2.1 (0.7, 4.1) years for denosumab (also higher in women vs men: 2.3 [0.7, 4.3] vs. 1.0 [0.0, 2.8] years).

**Fig. 2:**
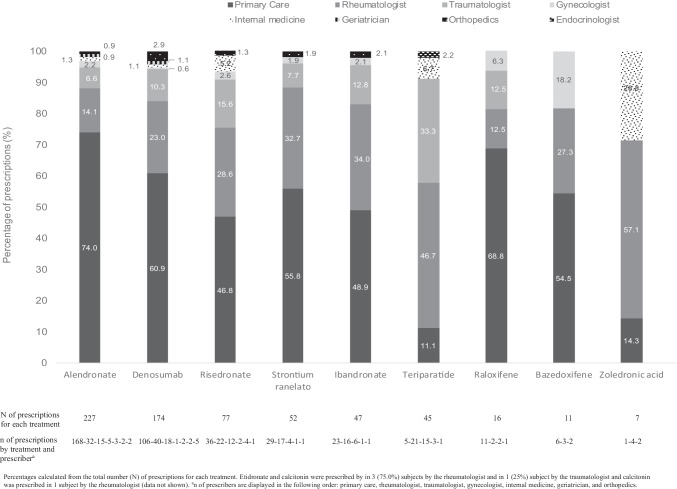
Prescribers of OP treatments—data from Spanish Primary Care

PC physicians were the most common prescribers of all OP treatments except teriparatide and zoledronate, for whom rheumatologists were the most common prescribers (Fig. 2).

**Fig. 3:**
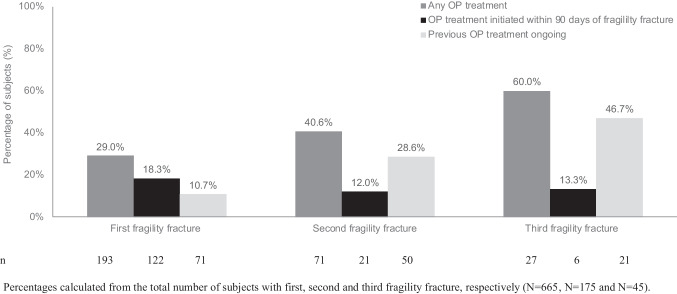
Subjects receiving OP treatment following first, second and third fragility fracture

Figure 3 summarizes OP treatment rates after the first, second and third fragility fracture. Subjects with one fracture had a lower treatment rate than those with multiple fractures. Only 18% of subjects initiated treatment within 90 days of a first fracture, falling to 12% and 13% after a second and third fracture, respectively. Among subjects with a second fracture, over one-quarter were continuing prior OP treatment at the time of their second fracture; and among subjects with a third fracture, almost half were continuing prior OP treatment at the time of their third fracture. Compared with women, men were less likely to receive treatment or continue previous treatment after their first fragility fracture (21.4% (21/98) vs 30.3% (172/567) and 2.0% (2/98) vs 12.2% (69/567), respectively).

### OP follow-up visits and diagnostic test used

**Fig. 4:**
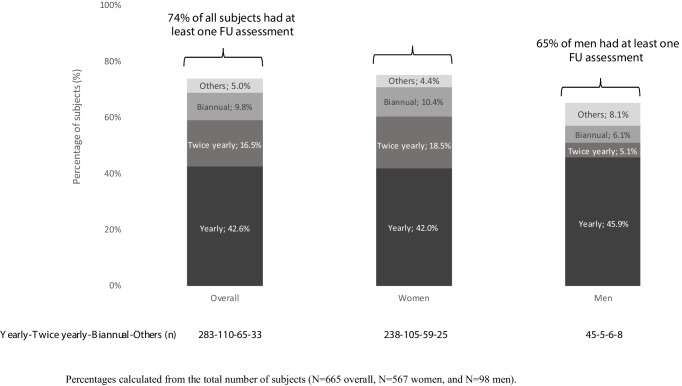
Subjects with post-fracture follow-up visits (overall and by sex)

Over one-quarter (174/665 [26.2%]) of subjects with a fragility fracture did not have follow-up visits post-fracture. Men were less likely to have follow-up visits than women (no follow-up visits: 34/98 [34.7%] vs 140/567 [24.7%]). The frequency of follow-up visits is summarized in Fig. 4. The most common schedule of OP follow-up visits was yearly, followed by half-yearly and biennial, with a similar trend in men and women. Older subjects (≥ 80 years) were more likely to have yearly follow-up visits (164/377 [43.5%]) than younger subjects (48/116 [41.4%] and 71/172 [41.3%] in subjects aged 70–74 and 75–79 years, respectively) and less likely to have biennial visits (32/377 [8.5%] vs 14/116 [12.1%] and 19/172 [11.0%], respectively).

The most commonly used follow-up tests were blood tests (85.9% [571/665] of subjects), radiography (48.4% [322/665]) and densitometry (39.2% [261/665]). There was no difference in rates of follow-up tests between men and women': blood tests, 86.1% (488/567) of women and 84.7% (83/98) of men; radiography, 50.3% (285/567) and 37.8% (37/98), respectively; densitometry, 41.6% (236/567) and 25.5% (25/98). Densitometry tests were more likely to be performed in younger subjects, being performed in half (50.0% [58/116]) of subjects aged 70–74 years vs 44.2% (76/172) and 33.7% (127/377) of those aged 75–79 and ≥ 80 years, respectively. Radiography tests were less common among younger subjects (41.4% [48/116] of subjects aged 70–74 years vs 51.2% [88/172] and 49.3% [186/377] of those aged 75–79 and ≥ 80 years, respectively).

## Discussion

We report real-world data regarding the clinical and therapeutic management of secondary fracture prevention by PC physicians in 665 subjects aged 70 years or over. Subjects in our study had a mean age of 82 years and had typically experienced only one fragility fracture. The first fracture was reported at a mean age of 76 years and in most (78%) cases was related to a fall, with similar results by sex, except for a shorter time between first and second fragility fracture in men.

The most commonly prescribed first-line OP treatments were alendronate followed by denosumab, with treatment patterns similar in men and women. A similar trend was observed in a cross-sectional study of women aged ≥ 70 years seen in PC across 8 European countries that reported a higher use of bisphosphonates compared to denosumab as first-line OP treatment (66% [58% oral bisphosphates and 8% parenteral bisphosphonates] vs 28%) [14]. The median duration of alendronate treatment in our study was slightly higher than for denosumab (2.5 and 2.1 years, respectively). It has been suggested a drug holiday for bisphosphonates of 3 to 5 years as they are retained in bone for extended periods with extended anti-fracture effects after discontinuation. However, the optimal duration is undetermined and should be re-evaluated after 1–2 years, depending on BF used [15]. Specifically, treatment with alendronate has shown maintenance of bone density among subjects who discontinued after 5 years [16]. Conversely, the bone-maintaining effects of denosumab wear off soon after stopping [17], with subjects not receiving subsequent OP medication experiencing 5–11% loss in bone mineral density in the first year after denosumab is stopped. Compared with denosumab, we observed a higher discontinuation rate among subjects treated with alendronate (42% vs 16%), possibly due to concerns regarding the potential side effects of bisphosphonates, including osteonecrosis of the jaw and/or atypical subtrochanteric femur fractures [18]. Following treatment discontinuation, bone mineral density (BMD) and fracture risk can be expected to return to baseline or worse slowly, in the case of bisphosphonates, or quickly, in the case of non-bisphosphonates [19]. This highlights the important role that specialists and primary care physicians play in increasing adherence, monitoring patients with bone markers and BMD testing and providing adequate instructions, feedback and support, and educational materials and sessions [20].

Treatment guidelines recommend the effect of OP treatment be confirmed by BMD and the absence of new fracture [13], therefore follow-up visits in subjects with OP are vital. However, over one-quarter of subjects in our study did not have follow-up visits, with the most common schedule of OP follow-up visits being yearly. While there is no clear evidence to guide the frequency of follow-up recommendations following a diagnosis of OP and treatment initiation, it is reasonable to assess response, adherence and tolerability to therapy at least once, after no less than 24 months [21].

In general, the most common prescribers of OP treatment in our study were PC physicians, followed by rheumatologists and traumatologists, a finding which is consistent with other studies conducted in the PC setting [22, 23]. As expected, teriparatide and zoledronate were most commonly prescribed in a hospital setting, Consistent with previous studies, gynecologists were most likely to prescribe bazedoxifene, followed by raloxifene [23].

While a number of effective and well tolerated OP treatments are available in clinical practice, the OP treatment gap has remained stable or increased in recent years [24]. It is important to highlight the high OP treatment gap we observed in Spanish PC, with over half (57%) of subjects not receiving OP treatment at study entry despite having suffered a fragility fracture. Our results are consistent with previous studies which have reported a treatment gap between 53 to 91% [14, 24, 25]. Moreover, OP diagnosis reduced the treatment gap (at study entry) from 87% in subjects without a diagnosis to 41% in those with a diagnosis^9^ (74% vs 21% treatment gap at any time during the study). Similarly, a retrospective cross-sectional study of women aged ≥ 70 years seen in PC across 8 European countries reported a treatment gap of 94% in subjects without an OP diagnosis vs 31% in those with a diagnosis [14]. In addition, we observed a higher treatment gap (at study entry) in men compared with women (71% vs 54%), consistent with 2014–2015 data from secondary fracture prevention in Europe [24]. However, a European cohort study of secondary fracture prevention subjects reported a higher treatment gap in women vs men (overall, 73% vs 63%; being higher in women vs men in France, Italy, Spain, Sweden and the UK, while it was maintained similar between sex in Germany [8]. Among subjects in Sweden untreated within one year of fracture, the same study reported the treatment gap in treatment naïve subjects to be higher for men vs women (96% vs 89%), and the treatment gap in subjects with prior treatment to be slightly lower in men vs women (47% vs 52%). Thus, studies about treatment gap by sex showed inconsistent results.

It should be noted that the treatment gap was higher among subjects with a single fracture than those with multiple fractures. This fact could be explained because these are the group of subjects with a much higher risk of fracture. According to these results, we could suggest that an improvement of early diagnostic, OP management and follow-up of subjects in secondary fracture prevention in PC is needed to reduce the number of patients at higher risk.

Almost half of the subjects in our study discontinued OP treatment, mostly by decision of the treating specialist/investigator or subject, or due to tolerability problems or non-compliance. Several factors could have contributed to this high discontinuation rate, including concerns about rare side effects, comorbidities and inadequate long-term efficacy. Indeed, ineffectiveness of anti-resorptive drugs has been associated with an increase in the number of untreated subjects [11]. Possible factors contributing to non-compliance include difficulty in perceiving the benefits of therapy, side effects, medication costs, lack of patient motivation and/or education. In a retrospective cohort study of women aged ≥ 50 years receiving OP treatment in Spain; 1-year persistence ranged from 35.8% (strontium ranelate) to 65.8% (denosumab), before dropping to only 10.3% (strontium ranelate) to 45.4% (denosumab) by 2 years. Compared to alendronate, the risk of discontinuation within 1 year was lower for teriparatide and denosumab, and only denosumab showed a reduced risk of discontinuation at 2-years [26]. Reported adherence to OP treatment varies widely, according to the method used. For example, when assessed using the Morisky–Green questionnaire, a study from Spanish PC reported over two-thirds (68.7%) of women to be non-compliant compared with 11.2% when adherence was self-reported by the study participants [27]. Related to this, Martínez-Laguna et al. [28] reported that combining the Morisky–Green questionnaire and determination of the carboxyterminal telopeptide of type I collagen allowed better assessment [29]. While the International Osteoporosis Foundation and European Calcified Tissue Society Working Group advocate the use of bone remodeling markers to assess adherence to bisphosphonates [30], these are rarely utilised in PC. While difficult and complex to measure in clinical practice [27], improving adherence, and, in turn, persistence, is vital for treatment effectiveness. Related to this, Yeh et al. [10] estimated the impact of improvements in risk assessment and/or treatment adherence on the economic burden of OP across eight European countries They estimated that 50% increases in treatment adherence and fracture risk assessment would result in an 18% reduction in fracture incidence and a 15% reduction in net costs (saving of € 2.8 billion) by 2040. Similarly, 10% increases in treatment adherence and fracture risk assessment could result in an 8.4% reduction in fracture incidence and a 7.6% reduction in net costs (saving of € 1.4 billion) by 2040. Actions to improve adherence include patient education with counselling, adherence monitoring with feedback and dose simplification (consisting of flexible dosing regimen) [31].

This study has strengths and limitations. For example, the inclusion of centers from different Spanish regions provides a more representative sample of the population in Spain. By contrast, the observational and retrospective study design contribute to missing data and is a limitation. Moreover, the small sample of subjects in some subgroup analysis is also a limitation. Finally, the PC centers that participated tended to have high OP experience which could bias the treatment rates compared to PC centers without OP experience.

## Conclusions

This observational study reports suboptimal secondary fracture prevention in Spanish primary care, with a large treatment gap in elderly subjects experiencing fragility fracture, low treatment persistence among subjects who do receive treatment, and more than one-quarter having no follow-up visits post-fracture. These data highlight the need to improve secondary fracture prevention in primary care by enhancing the early diagnostic, assessment of risk of fractures, management of OP treatment, adherence, and follow-up of OP subjects specially in subgroups with higher risk factors as those with multiple fractures.

## Supplementary Information

Below is the link to the electronic supplementary material.

Supplementary file1 (DOCX 24 KB)

## Data Availability

Qualified researchers may request data from Amgen clinical studies. Complete details are available at the following: https://wwwext.amgen.com/science/clinical-trials/clinicaldata-transparencypractices/clinical-trial-data-sharing-request/
