# Supplementary material for: Secondary fracture prevention in Spanish primary care: results of the PREFRAOS Study
Source: Arch Osteoporos. 2024 May 9;19(1):35. doi: 10.1007/s11657-024-01394-3 (PMC11081989; doi:10.1007/s11657-024-01394-3)
Supplement: Supplementary file 1 — Supplementary file1 (DOCX 24 KB) [file 11657_2024_1394_MOESM1_ESM.docx]

**Supplementary Methods Information**

The following variables were obtained retrospectively from subjects’ medical records:

1) sociodemographic and clinical variables: age, sex, age at first fracture, time from first fracture, first fracture related to a fall, and number of fragility fractures.

2) risk factors for fracture at the time of data collection (or last information available in the medical records): body mass index [BMI], history of falls, history of parental hip fracture, current smoking, alcohol intake ≥3 units/day, rheumatoid arthritis, secondary OP (all diseases considered in the FRAX^©^ tool) and associated medications (oral glucocorticoids, aromatase inhibitors, GnRH analogues, anticonvulsants, proton-pump inhibitors, antihypertensive drugs and statins).

3) OP treatment (type of drug, reasons of discontinuation, treatment duration, and treatment prescription by specialist). Prior OP treatment was defined as the OP treatment received and ended before informed consent. Current OP treatment was defined as the OP treatment received (ongoing or initiated) at informed consent and OP treatment at any time was defined as prior OP treatment or current OP treatment.

4) OP follow-up visits and tests conducted.

**Supplementary Table SI1.** Discontinuation of OP treatments and reasons by sex

|  | | | **Treatments in women (N=608)** | | **Treatments in men (N=53)** | | **Total treatments (N=661)** | |  |
| --- | --- | --- | --- | --- | --- | --- | --- | --- | --- |
| **Total number of prescriptions** | | | | | | | | |  |
| **Alendronate** | | | | | | | | |  |
|  | Number of prescriptions | n (%) | | 212 (34.9%) | | 15 (28.3%) | | 227 (34.3%) | |
|  | Number of treatments discontinued | n (%) | | 94 (44.3%) | | 2 (13.3%) | | 96 (42.3%) | |
|  | ----- Reason for discontinuation, n (%) | Tolerability problems | | 19 (20.2%) | | 2 (100.0%) | | 21 (21.9%) | |
|  |  | Lack of efficacy | | 8 (8.5%) | |  | | 8 (8.3%) | |
|  |  | Non-compliance | | 4 (4.3%) | |  | | 4 (4.2%) | |
|  |  | Dental procedures or surgeries | | 2 (2.1%) | |  | | 2 (2.1%) | |
|  |  | Investigator's decision | | 17 (18.1%) | |  | | 17 (17.7%) | |
|  |  | Specialist's decision | | 23 (24.5%) | |  | | 23 (24.0%) | |
|  |  | Subject's decision | | 21 (22.3%) | |  | | 21 (21.9%) | |
|  | **Denosumab** |  | |  | |  | |  | |
|  | Number of prescriptions | n (%) | | 159 (26.2%) | | 15 (28.3%) | | 174 (26.3%) | |
| \| Number of treatments discontinued \| n (%) \| 23 (14.5%) \| 4 (26.7%) \| 27 (15.5%) \| \| --- \| --- \| --- \| --- \| --- \| | | | | | | | | |  |
| \| ----- Reason for discontinuation, n (%) \| Tolerability problems \|  \| 1 (25.0%) \| 1 (3.7%) \| \| --- \| --- \| --- \| --- \| --- \| | | | | | | | | |  |
| \|  \| Non-compliance \| 2 (8.7%) \| 1 (25.0%) \| 3 (11.1%) \| \| --- \| --- \| --- \| --- \| --- \| | | | | | | | | |  |
| \|  \| Investigator's decision \| 2 (8.7%) \|  \| 2 (7.4%) \| \| --- \| --- \| --- \| --- \| --- \| | | | | | | | | |  |
| \|  \| Specialist's decision \| 4 (17.4%) \|  \| 4 (14.8%) \| \| --- \| --- \| --- \| --- \| --- \| | | | | | | | | |  |
| \|  \| Subject's decision \| 15 (65.2%) \| 2 (50.0%) \| 17 (63.0%) \| \| --- \| --- \| --- \| --- \| --- \| | | | | | | | | |  |
| **Risedronate** | | | | | | | | |  |
|  | Number of prescriptions | n (%) | | 68 (11.2%) | | 9 (17.0%) | | 77 (11.6%) | |
|  | Number of treatments discontinued | n (%) | | 44 (64.7%) | | 5 (55.6%) | | 49 (63.6%) | |
|  | ----- Reason for discontinuation, n (%) | Tolerability problems | | 11 (25.0%) | | 1 (20.0%) | | 12 (24.5%) | |
|  |  | Lack of efficacy | | 2 (4.5%) | |  | | 2 (4.1%) | |
|  |  | Non-compliance | | 1 (2.3%) | | 1 (20.0%) | | 2 (4.1%) | |
|  |  | Investigator's decision | | 8 (18.2%) | | 1 (20.0%) | | 9 (18.4%) | |
|  |  | Specialist's decision | | 15 (34.1%) | | 2 (40.0%) | | 17 (34.7%) | |
|  |  | Subject's decision | | 7 (15.9%) | |  | | 7 (14.3%) | |
| **Ibandronate** | | | | | | | | |  |
|  | Number of prescriptions | n (%) | | 45 (7.4%) | | 2 (3.8%) | | 47 (7.1%) | |
|  | Number of treatments discontinued | n (%) | | 24 (53.3%) | | 2 (100.0%) | | 26 (55.3%) | |
|  | ----- Reason for discontinuation, n (%) | Tolerability problems | | 7 (29.2%) | |  | | 7 (26.9%) | |
|  |  | Non-compliance | | 1 (4.2%) | |  | | 1 (3.8%) | |
|  |  | Dental procedures or surgeries | | 1 (4.2%) | |  | | 1 (3.8%) | |
|  |  | Investigator's decision | | 4 (16.7%) | | 1 (50.0%) | | 5 (19.2%) | |
|  |  | Specialist's decision | | 9 (37.5%) | | 1 (50.0%) | | 10 (38.5%) | |
|  |  | Subject's decision | | 2 (8.3%) | |  | | 2 (7.7%) | |
| **Raloxifene** | | | | | | | | |  |
|  | Number of prescriptions | n (%) | | 16 (2.6%) | | 0 (0.0%) | | 16 (2.4%) | |
|  | Number of treatments discontinued | n (%) | | 13 (81.3%) | |  | | 13 (81.3%) | |
|  | ----- Reason for discontinuation, n (%) | Tolerability problems | | 1 (7.7%) | |  | | 1 (7.7%) | |
|  |  | Non-compliance | | 3 (23.1%) | |  | | 3 (23.1%) | |
|  |  | Specialist's decision | | 5 (38.5%) | |  | | 5 (38.5%) | |
|  |  | Subject's decision | | 4 (30.8%) | |  | | 4 (30.8%) | |
| **Bazedoxifene** | | | | | | | | |  |
|  | Number of prescriptions | n (%) | | 11 (1.8%) | | 0 (0.0%) | | 11 (1.7%) | |
|  | Number of treatments discontinued | n (%) | | 9 (81.8%) | |  | | 9 (81.8%) | |
|  | ----- Reason for discontinuation, n (%) | Lack of efficacy | | 2 (22.2%) | |  | | 2 (22.2%) | |
|  |  | Investigator's decision | | 2 (22.2%) | |  | | 2 (22.2%) | |
|  |  | Specialist's decision | | 4 (44.4%) | |  | | 4 (44.4%) | |
|  |  | Subject's decision | | 1 (11.1%) | |  | | 1 (11.1%) | |
| **Strontium ranelate** | | | | | | | | |  |
|  | Number of prescriptions | n (%) | | 50 (8.2%) | | 2 (3.8%) | | 52 (7.9%) | |
|  | Number of treatments discontinued | n (%) | | 41 (82.0%) | | 2 (100.0%) | | 43 (82.7%) | |
|  | ----- Reason for discontinuation, n (%) | Tolerability problems | | 12 (29.3%) | |  | | 12 (27.9%) | |
|  |  | Non-compliance | | 2 (4.9%) | |  | | 2 (4.7%) | |
|  |  | Investigator's decision | | 10 (24.4%) | | 1 (50.0%) | | 11 (25.6%) | |
|  |  | Specialist's decision | | 11 (26.8%) | |  | | 11 (25.6%) | |
|  |  | Subject's decision | | 6 (14.6%) | | 1 (50.0%) | | 7 (16.3%) | |
| **Teriparatide** | | | | | | | | |  |
|  | Number of prescriptions | n (%) | | 40 (6.6%) | | 5 (9.4%) | | 45 (6.8%) | |
|  | Number of treatments discontinued | n (%) | | 23 (57.5%) | | 3 (60.0%) | | 26 (57.8%) | |
|  | ----- Reason for discontinuation, n (%) | Tolerability problems | | 2 (8.7%) | |  | | 2 (7.7%) | |
|  |  | Lack of efficacy | | 1 (4.3%) | |  | | 1 (3.8%) | |
|  |  | Non-compliance | | 1 (4.3%) | | 1 (33.3%) | | 2 (7.7%) | |
|  |  | Investigator's decision | | 1 (4.3%) | |  | | 1 (3.8%) | |
|  |  | Specialist's decision | | 16 (69.6%) | | 2 (66.7%) | | 18 (69.2%) | |
|  |  | Subject's decision | | 2 (8.7%) | |  | | 2 (7.7%) | |
| **Zoledronic acid** | | | | | | | | |  |
|  | Number of prescriptions | n (%) | | 4 (0.7%) | | 3 (5.7%) | | 7 (1.1%) | |
|  | Number of treatments discontinued | n (%) | | 2 (50.0%) | | 2 (66.7%) | | 4 (57.1%) | |
|  | ----- Reason for discontinuation, n (%) | Specialist's decision | | 2 (100.0%) | | 1 (50.0%) | | 3 (75.0%) | |
|  |  | Subject's decision | |  | | 1 (50.0%) | | 1 (25.0%) | |
| **Etidronate** | | | | | | | | |  |
|  | Number of prescriptions | n (%) | | 3 (0.5%) | | 1 (1.9%) | | 4 (0.6%) | |
|  | Number of treatments discontinued | n (%) | | 3 (100.0%) | | 1 (100.0%) | | 4 (100.0%) | |
|  | ----- Reason for discontinuation, n (%) | Non-compliance | | 3 (100.0%) | | 1 (100.0%) | | 4 (100.0%) | |
| **Calcitonin** | | | | | | | | |  |
|  | Number of prescriptions | n (%) | | 0 (0.0%) | | 1 (1.9%) | | 1 (0.2%) | |
|  | Number of treatments discontinued | n (%) | |  | | 1 (100.0%) | | 1 (100.0%) | |
|  | ----- Reason for discontinuation, n (%) | Tolerability problems | |  | | 1 (100.0%) | | 1 (100.0%) | |
